# Effects of Ti on the Microstructural Evolution and Mechanical Property of the SiBCN-Ti Composite Ceramics

**DOI:** 10.3390/ma16093560

**Published:** 2023-05-06

**Authors:** Hao Peng, Daxin Li, Zhihua Yang, Wenjiu Duan, Dechang Jia, Yu Zhou

**Affiliations:** 1 Institute for Advanced Ceramics, School of Materials Science and Engineering, Harbin Institute of Technology, Harbin 150001, China; penghao211@163.com (H.P.); zhyang@hit.edu.cn (Z.Y.); zhouyu@hit.edu.cn (Y.Z.); 2 Key Laboratory of Advanced Structural-Functional Integration Materials & Green Manufacturing Technology, Harbin Institute of Technology, Harbin 150001, China; 3 State Key Laboratory of Advanced Welding and Joining, Harbin Institute of Technology, Harbin 150001, China; 4 Tsinghua Shenzhen International Graduate School, Tsinghua University, Shenzhen 518055, China; dwenjiu@foxmail.com

**Keywords:** SiBCN, Ti, microstructural evolution, mechanical properties, mechanical alloying

## Abstract

In this study, amorphous + nanocrystalline Ti-BN mixed powders were obtained through first-step mechanical alloying; subsequently, almost completely amorphous SiBCN-Ti mixed powders were achieved in the second-step milling. The SiBCN-Ti bulk ceramics were consolidated through hot pressing sintering at 1900 °C/60 MPa/30 min, and the microstructural evolution and mechanical properties of the as-sintered composite ceramics were investigated using SEM, XRD, and TEM techniques. The as-sintered SiBCN-Ti bulk ceramics consisted of substantial nanosized BN(C), SiC, and Ti(C, N) with a small amount of Si_2_N_2_O and TiB_2_. The crystallized BN(C) enwrapped both SiC and Ti(C, N), thus effectively inhibiting the rapid growth of SiC and Ti(C, N). The sizes of SiC were ~70 nm, while the sizes of Ti(C, N) were below 30 nm, and the sizes of Si_2_N_2_O were over 100 nm. The SiBCN-20 wt.% Ti bulk ceramics obtained the highest flexural strength of 394.0 ± 19.0 MPa; however, the SiBCN-30 wt.% Ti bulk ceramics exhibited the optimized fracture toughness of 3.95 ± 0.21 GPa·cm^1/2^, Vickers hardness of 4.7 ± 0.27 GPa, Young’s modulus of 184.2 ± 8.2 GPa, and a bulk density of 2.85 g/cm^3^. The addition of metal Ti into a SiBCN ceramic matrix seems to be an effective strategy for microstructure optimization and the tuning of mechanical properties, thus providing design ideas for further research regarding this family of ceramic materials.

## 1. Introduction

With the rapid development of aerospace, metallurgy, energy, and information technology, basic scientific research concerning high-temperature wear-resistant materials has become a major topic in materials science. For example, the cylinder walls and sealing parts of aero-engines, which are typically exposed to extreme high-temperature conditions, require structural materials with high strength, a satisfying fracture toughness, and good resistance to oxidation, thermal shock, and ablation to ensure the safety and reliability of structural parts exposed to harsh environments [1,2,3,4].

SiBCN-based ceramics have attracted intense attention in the field of high-temperature structural materials owing to their low density, good mechanical properties, desirable thermal shock resistance, oxidation resistance, and high-temperature creep resistance [5,6,7]. Generally, the SiBCN-typed ceramics were prepared mainly through the polymer/precursor-derived ceramics (PDCs) route and the mechanical alloying (MA) method., By using organic precursors as raw materials, the SiBCN PDCs were first cross-linked under an inert atmosphere at ~200–400 °C for a long period. During the pyrolysis process, the ceramic precursors gradually disintegrated, removing small molecules, and finally, solidified to form covalently bonded SiBCN ceramics. For the resulting SiBCN PDCS, the four elements are distributed uniformly, leading to excellent chemical stability with an amorphous nature up to 1700 °C under an air environment [8]. The main advantage of SiBCN PDCs is that their final chemistry and microstructure can be tailored at the atomic scale by choosing reasonable monomers, functional groups, and synthesis parameters. According to previous studies, the incorporation of ultra-high-temperature ceramics (UHTCs) in a SiBCN ceramic matrix is considered to be an effective strategy for improving the mechanical and high-temperature properties of SiBCN ceramics [9,10]. The microstructural evolution and thermal stability of porous PDC ZrB_2_-SiBCN and HfN-SiBCN composite ceramics have been explored [11,12]. However, the mechanical property aspects of these ceramics have not been investigated due to limitations in terms of sample size. However, the organic polymers/precursors used were expensive and toxic, which are environmentally unfriendly [13,14]. Carefully monitoring gas release and mass loss during pyrolysis is necessary to avoid porous structures and to carefully control shrinkage (even microcracking) [15]. Although the shrinkage problems during pyrolysis can be improved through the addition of active fillers (such as ceramics or metal powers), these issues limit the use of the PDC method for the preparation of dense SiBCN ceramics as well as structural component applications [16,17]. Therefore, the development of a new method for the preparation of SiBCN ceramics is inevitable.

Recently, SiBCN ceramics that have the targeted composition have been fabricated using the mechanical alloying method [18]. In recent years, mechanical alloying rapidly developed as a technique and has been widely used to prepare dispersion-reinforced materials, intermetallic compounds, amorphous/nanocrystalline, supersaturated solid solutions, and other metallic and ceramic materials. The mechanical alloying technology uses a wide range of raw materials, and the process is simple enough to produce non-equilibrium phases and nanophases that cannot be prepared through the use of conventional methods. During the mechanical alloying process, large amounts of mechanical energy are impacted on the mixing powders within a short period to cause repeated welding, fracturing, and re-welding, finally obtaining amorphous SiBCN powders. The raw materials used in mechanical-alloying-derived SiBCN ceramics are inexpensive, non-toxic, and less hazardous to the environment and operators. 

The merit of the mechanical alloying method is in the preparation of dense bulk SiBCN ceramics and composites on a large scale (tens of centimeters) [19,20,21]. For example, SiBCN composites with the addition of MWCNTs, graphene, Zr, Al [22], AlN, LaB_6_ [23], HfC + TaC, C_f_, SiC_f_ [24], etc., exhibited improved mechanical properties compared to pure SiBCN under the same sintering conditions (Table 1). For instance, SiBCN-Al ceramics produced through mechanical alloying with the addition of aluminum (Al) into SiBCN exhibited satisfying flexural strength and fracture toughness [18]. The layered structure of LaB_6_ achieved through mechanical alloying also enabled desirable mechanical properties compared with pure SiBCN [20]. However, during the sintering process, some LaB_6_ reacted with the SiBCN ceramic matrix to generate La_2_B_2_C_6_, and consequently, the strengthening and toughening effects of LaB_6_ were significantly reduced. In another contribution, the addition of SiC_f_ into the SiBCN matrix resulted in decreased mechanical strength due to fiber degradation and strong interfacial bonding [24]. In all cases, these SiC_f_/SiBCN composites consolidated at 1900 °C showed brittle fracture with few degree-bridged fiber and fiber pull-out.

Ti(C, N)-based ceramics, as one of the most promising materials in terms of semi-finishing and finishing cutting tools for steel and cast iron, have attracted extensive research attention due to their high hardness, good thermal stability, and good resistance to creep and wear [25]. However, the wide application of Ti(C, N) ceramics when under high-temperature conditions is restricted due to their poor thermal shock resistance and oxidation resistance [26]. Therefore, with the combination of the advantages of SiBCN and Ti(C, N) ceramics, the preparation of SiBCN-Ti composite ceramics with high mechanical performance with satisfying microstructures is expected. 

In this study, the base amorphous SiBCN-Ti powders were prepared through the use of mechanical alloying using two-step ball milling. Subsequently, nano SiBCN bulk ceramics with in situ formed SiC, BN(C) and Ti(C, N) nanocrystals were consolidated by using the hot-pressing sintering technique. The motivation of this study was to develop an understanding of the microstructural evolution and the mechanical property relationship in these composite ceramics as a function of the added Ti content.

## 2. Materials and Methods

### 2.1. Preparation of Amorphous SiBCN-Ti Powders

In this work, the base amorphous SiBCN-Ti powders were prepared using a Fritsch P4 high-energy ball-miller. The raw materials used were cubic silicon powders (c-si, particle size 15.5 μm, Si content > 99%, Beijing Mengtai Research and Technology Development Center, Beijing, China), hexagonal boron nitride (h-BN, particle size 0.6 μm, BN content > 98%, Shandong Qingzhou Fangyuan Boron Nitride Plant, Qingdao, China), graphite powders (C, particle size 5 μm, C content > 99%, Qingdao Huatai Lubrication and Sealing Technology Co., Ltd., Qingdao, China), and titanium metal powders (Ti, particle size 15 μm, Ti content > 99.99%, Shanghai Maclean Biochemical Technology Co., Ltd., Shanghai, China). Firstly, metal Ti and h-BN powders with a molar ratio of 3:2 were loaded into the ball-milling tanks for 1–30 h of ball-milling (with a ball-to-material ratio of 20:1). Then, the c-Si, graphite, and residual h-BN powders (where mole ratio of h-BN:c-Si:graphite = 1:2:3) were subsequently loaded into the ball-mill tanks for a further milling of 20 h. The Ti in the base amorphous SiBCN-Ti powders was designed to 5, 10, 15, 20, and 30 wt.%. All of the steps were conducted in a glove box filled with high-purity argon gas (Harbin Tianci Gas Distribution Co., Ltd., Harbin, China) to prevent possible oxidation from occurring. The main disc speed was set as 350 r/min, and the planetary disc speed was 700 r/min. To prevent the machine from overheating, the milling program was set to take a 10 min break every 50 min of operation.

### 2.2. Preparation of Nanocrystalline SiBCN-Ti Bulk Ceramics

The as-milled SiBCN-Ti ceramic powders were poured into the graphite molds with an inner diameter of 36 mm. The hot-pressing sintering was operated at 1900 °C/60 MPa/30 min under one bar of N_2_. The heating rate was set to 30 °C/min for a temperature below 1200 °C; beyond this, the heating rate was held at 25 °C/min. The axial pressure was loaded once the temperature reached 1200 °C. After sintering, the furnace cooled down naturally. When the temperature decreased to 1200 °C, the pressure was unloaded completely. The as-sintered samples were labeled as Ti-5, Ti-10, Ti-15, Ti-20, and Ti-30, respectively. 

### 2.3. Characterization Methods

The performance tests of the specimens were performed on a full-scale mechanical testing machine (Instron 5569, USA). The flexural strength and Young’s modulus were obtained from 3 mm × 4 mm × 20 mm bars using the three-point bending testing method with a span of 16 mm and an indenter speed of 0.5 mm/min. The fracture toughness was determined using the single-sided notched beam method with the specimen bar size of 2 mm × 4 mm × 20 mm and a notch depth of 2 mm. Vickers hardness was measured in an HVS-5 (Laizhou Huayin Testing Instruments Co., Ltd., China) machine by polishing the surface of the specimen, using a pressure of 15 kg, and holding it for 15 s. The bulk density of the samples was measured using the Archimedes drainage method. The measured samples were cleaned ultrasonically in ethanol and then dried, and the masses were weighed on a precise analytical balance with an accuracy of one part in ten thousand.

The phase composition of the specimens was measured by using an X’PERT X-ray diffractometer purchased from the Netherlands, Panalytical, with a scanning speed of 10° per minute and a scanning range of 10~90°. The microstructural/morphological evolution of the resulting powders and bulk ceramics was investigated through the use of a Hitachi su5000 scanning electron microscope and a field emission high-resolution transmission electron microscope (TEM, Talos F200x, 200 kV, FEI, USA.) coupled with an Oxford instrument UltimExtreme energy spectrum detector.

## 3. Results 

### 3.1. Synthesis and Characterization of SiBCN-Ti Powders 

**Figure 1:**
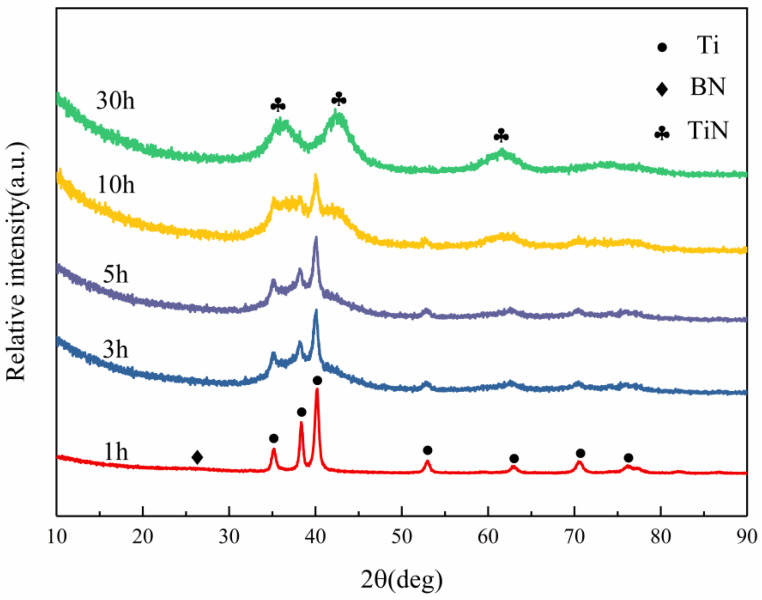
XRD patterns of the Ti and h-BN mixed powders with a molar ratio of 3:2 at different mechanical alloying times.

Figure 1 shows the XRD patterns of Ti and h-BN (molar ratio of Ti:BN = 3:2) mixed powders after undergoing ball milling for different time periods. After 1 h of ball milling, the h-BN diffraction peaks started to disappear significantly, while the diffraction peaks of Ti were still relatively obvious. After 3 h of ball milling, the h-BN diffraction peaks have completely disappeared, and after 10 h of ball milling, the intensity of Ti diffraction peaks has also decreased significantly, indicating that h-BN achieves solid-state amorphization easier than Ti. When the ball milling time was increased to 30 h, the XRD pattern presented with diffuse scattering peaks, representing the amorphous phase, indicative of both amorphous h-BN and Ti. However, broad peaks assigned to the TiN have been found. It seems that the composite powder did not experience phase transformation as the milling time was increased from 1 h to 10 h, but the Ti diffraction peaks shifted to smaller angles, clearly suggesting the increasing interplanar spacing, which may result from the insertion of the B and/or N atoms during high-energy ball milling. The degree of Ti lattice distortion increases gradually with the increasing milling up to 30 h, leading to the collapse of the Ti lattice, finally forming TiN. Since the initial molar ratio of the Ti:B is 3:2, it is not because the mass is too small that it is not detected. This should be accounted for by the presence of B-containing microcrystals whose size is too small to be identified using X-ray diffraction; therefore, it is necessary to observe the high-energy ball mill powder under high-resolution transmission electron microscopy to determine the degree of the powder amorphization.

**Figure 2:**
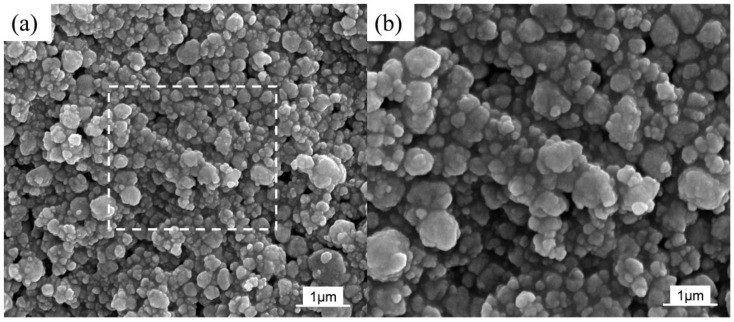
SEM images of Ti and h-BN mixed powders after 30 h of mechanical alloying: (**a**) low magnification; (**b**) high magnification of the white dashed area in the (**a**).

**Figure 3:**
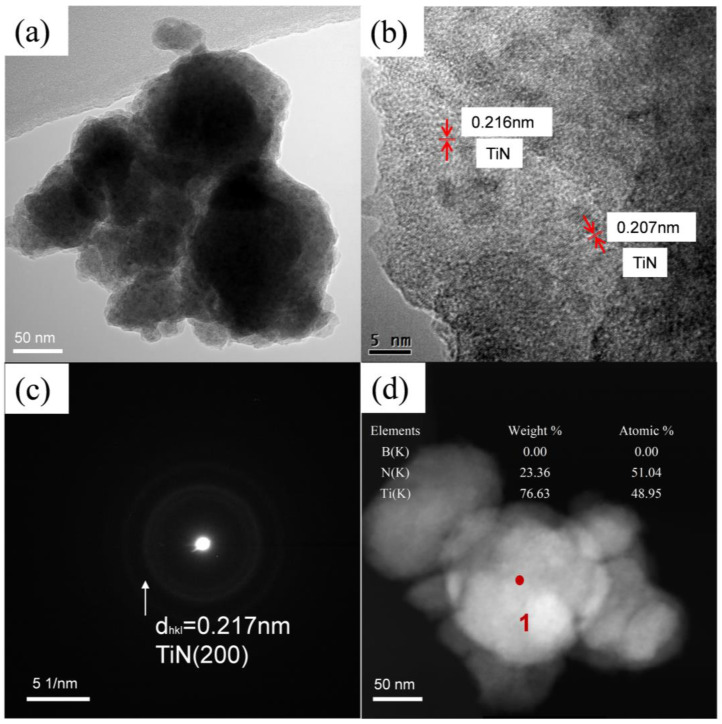
TEM and HRTEM images of Ti and h-BN mixed powder after 30 h of mechanical alloying: (**a**) TEM; (**b**) HRTEM; (**c**) corresponding SAED; (**d**) energy diffraction X-ray spectra was performed at the locations marked by red dots and numbers in the (**d**).

As shown in Figure 2, SEM was used to observe the morphologies and particle sizes of the as-milled Ti and h-BN mixed powders. It seems that the powders underwent an obvious hard agglomeration phenomenon due to the welding process. From Figure 2b, the size of the powder particle was around 200–500 nm. However, in HRTEM images (Figure 3), the size of the as-milled powder was about 100–200 nm. Thus, the so-called larger-sized particles were stacked through the agglomeration of smaller ones. Almost no nanocrystals appeared in the HRTEM images, suggesting the almost complete amorphization of Ti and BN mixed powders, which contrasted with the XRD results. 

**Figure 4:**
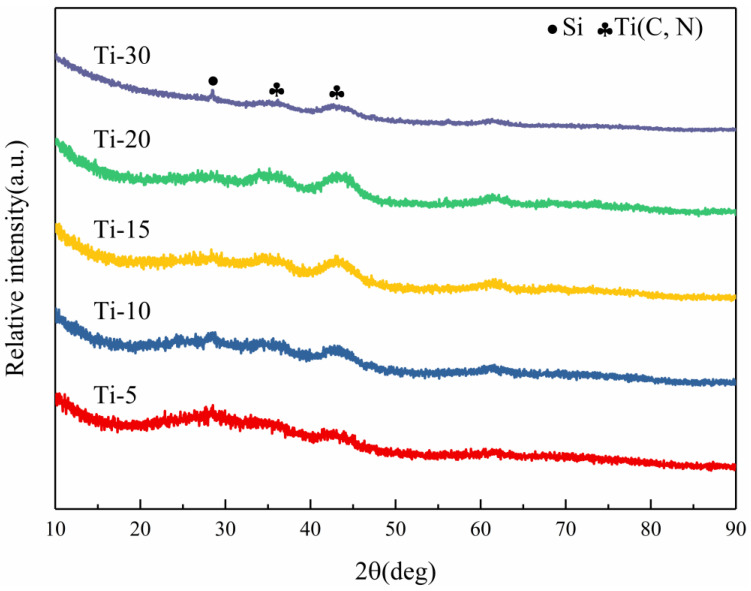
XRD patterns of SiBCN-Ti composite powders with different Ti content after 20 h of milling.

The XRD patterns of the as-milled SiBCN-Ti composite powders with different Ti contents are shown in Figure 4. For a Ti content below 30 wt.%, no trace of crystalline peaks was detected, indicating the complete destruction of the lattice structure to achieve solid-state amorphization. However, increasing the Ti content to 30 wt.% led to the emergence of new Ti(C, N) phases and Si. Actually, a few Ti(C, N) phases were also observed for SiBCN-Ti composite powders with 15 wt.% and 20 wt.% Ti addition since broad peaks assigned to Ti(C, N) appeared. With the increase in Ti introduction, the Ti(C, N) diffraction peak gradually enhanced. In the case of 30 wt.% Ti-addition, the appearance of a Si peak should be argued to be due to the inhibited amorphization process of Si; however, the detailed reasons for this unexpected result are still in their infancy.

**Figure 5:**
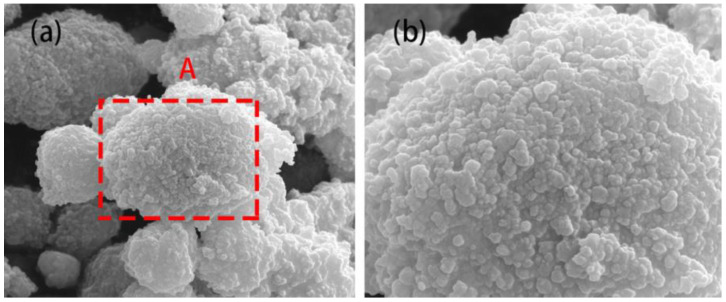
SEM images of SiBCN-15 wt.% Ti composite powders after mechanical alloying for 20 h: (**a**) low magnification; (**b**) high magnification of the red dashed area A in the (**a**).

**Figure 6:**
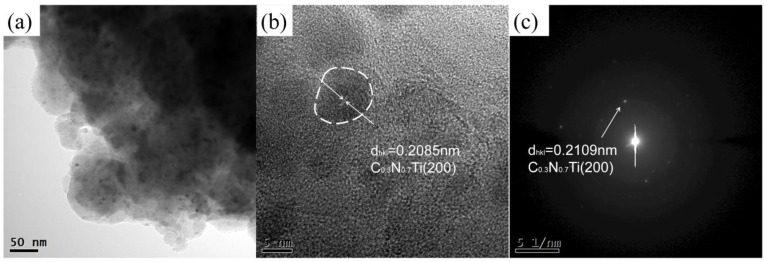
TEM image and HRTEM images of SiBCN-15 wt.% Ti composite powders after 20 h of ball milling: (**a**) TEM image; (**b**) HRTEM image, the white circle area is the Ti(C, N) nanocrystals; (**c**) corresponding SAED.

The morphologies and particle sizes of the SiBCN-Ti composite powders after 20 h of milling were observed using SEM. From Figure 5a, the size of SiBCN-Ti composite powders was around 1–5 μm; however, these larger size particles were formed through the agglomeration of smaller ones (Figure 5b). The TEM and HRTEM images of the SiBCN-15 wt.% Ti composite powders after 20 h of milling are shown in Figure 6. As indicated, the smaller particles were sized around 100–200 nm. Figure 6b clearly presents the formation of some nanocrystals in an amorphous matrix. The measured crystalline spacing of the nanocrystals was 0.2085 nm, close to the crystalline spacing of C_0.7_N_0.3_Ti (200) (Figure 6c). Thus, the ball-milling-driven nanocrystals, as confirmed both by XRD and HRTEM characterization, were those of C_0.7_N_0.3_Ti ~5–7 nm. 

During the mechanical alloying process, the raw material powder particles undergo a continuous cycle of deformation, crushing and cold sintering under a strong mechanical alloy process, leading to grain refinement and microstrain. With continuous mechanical alloying, increasing defects are generated on the surface of TiN particles. Larger microstrains and smaller grain sizes lead to higher dislocation density in the crystal. In addition, the reduction in grain size increases the surface area per unit volume. The higher surface energy provides the driving force for diffusion, resulting in the continuous solid solution of C atoms into TiN to finally generate Ti(C, N). However, because hard agglomerates are not easily deformed or broken, the pores between hard agglomerates are also difficult to be filled by ceramic particles during the sintering process of ceramic blanks, and the pores are difficult to exclude.

### 3.2. Microstructural Evolution and Mechanical Properties of SiBCN-Ti Bulk Ceramics 

**Figure 7:**
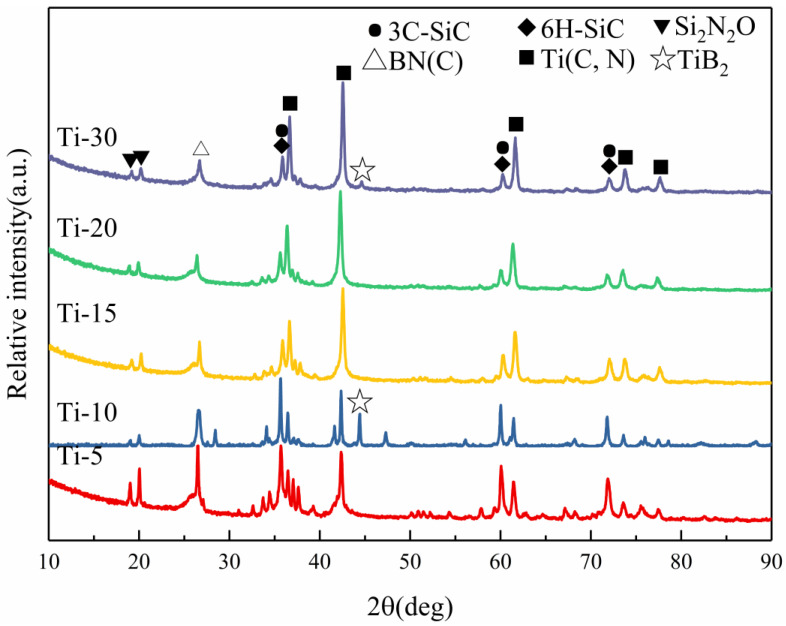
XRD patterns of SiBCN-Ti bulk ceramics after hot-press sintering at 1900 °C/60 MPa/30 min.

The phase composition of the as-sintered SiBCN-Ti bulk ceramics is shown in Figure 7. Obviously, the amorphous phases have crystallized into nanostructures, including the main phases of BN(C), SiC, and Ti(C, N), and the minority of Si_2_N_2_O and TiB_2_. Naturally, the diffraction peaks of the Ti(C, N) phases gradually enhanced with the increase in Ti content. 

**Figure 8:**
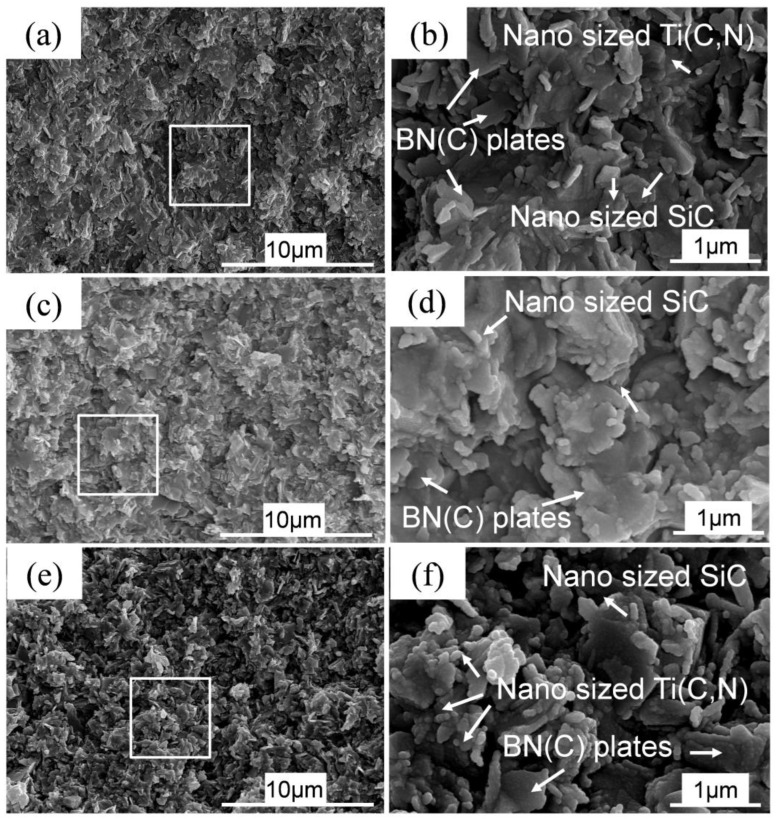
SEM fracture morphologies of SiBCN-Ti bulk ceramics after hot-press sintering at 1900 °C/60 MPa/30 min: (**a**,**b**) Ti-5, (**b**) shows an enlarged view of the white box area in (**a**); (**c**,**d**) Ti-15, (**d**) shows an enlarged view of the white box area in (**c**); (**e**,**f**) Ti-30, (**f**) shows an enlarged view of the white box area in (**e**).

**Figure 9:**
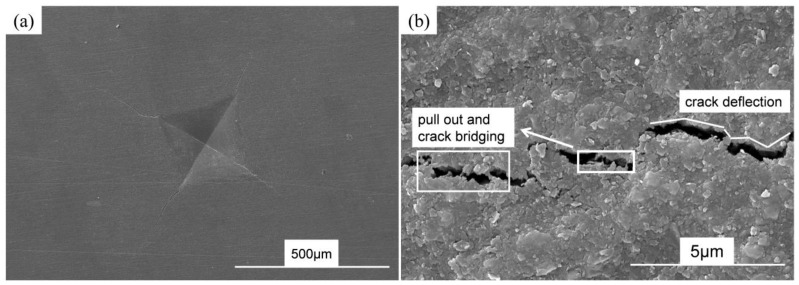
SEM microcrack morphologies of SiBCN-15 wt.% Ti bulk ceramics: (**a**) Indentation morphology; (**b**) microcracks deflection and bridging.

Figure 8 shows the fracture morphologies of the as-sintered SiBCN-Ti bulk ceramics with different Ti contents. As indicated, in all cases, the as-sintered bulk ceramics are dense without traces of microcracks and/or micro-holes. With lower Ti addition, the SiBCN-Ti composite particles were coarse, but the introduction of Ti refined the particles, which may benefit sintering densification and mechanical properties improvement. As a result, with the increase in Ti content, the bulk ceramics become denser. From magnifying the images, the BN(C) are in the form of a layered structure, while nanosized SiC and Ti(C, N) were distributed around the BN(C) phases. The formation of Si_2_N_2_O must facilitate the sintering densification during the high-pressure sintering process. In Figure 9, microcrack bridging, deflection, and BN(C) plate pull-out were frequently discovered in the as-sintered bulk ceramics.

**Figure 10:**
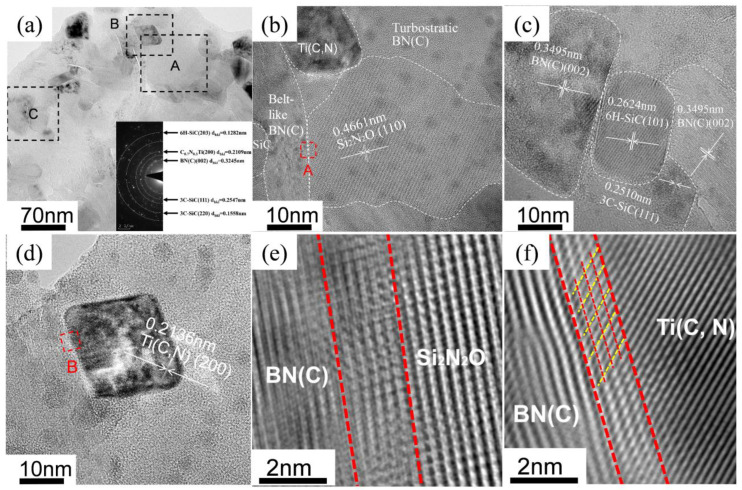
TEM analysis of SiBCN-15 wt.% Ti bulk ceramics: (**a**) BF-TEM image and corresponding SAED, regions A, B, and C in (**a**) correspond to (**b**–**d**), respectively; (**b**) HRTEM image of area A; (**c**) HRTEM image of area B; (**d**) HRTEM image of area C; (**e**) Fourier inverse transform of the red square dashed region A in (**b**); (**f**) Fourier inverse transform of the red square dashed region B in (**d**).

Further confirmed by TEM observation (Figure 10), SiC, BN(C), Ti(C, N) and Si_2_N_2_O were found. The SiCs were sized ~70 nm, while the sizes of Ti(C, N) were below 30 nm, and the sizes of Si_2_N_2_O were over 100 nm. The BN(C) was uniformly distributed between the nanosized SiC and Ti(C, N), forming a so-called capsule-like structure, which may be responsible for the slow growth of nano SiC and Ti(C, N). In fact, the BN(C) were in the form of belt-like and turbostratic structures. On the one hand, the belt-like BN(C) enwrapped the SiC grains inhibiting the short-range element diffusion, thus avoiding the abnormal growth of SiC. On the other hand, the turbostratic BN(C) also enwrapped Ti(C, N), further hindering the growth of Ti(C, N) and restraining the crystallization of BN©. Si_2_N_2_O was usually synthesized by reaction sintering a mixture of equimolar SiO_2_ and Si_3_N_4_ [27], or by a mixture of nitrided SiO_2_ and Si [28,29,30]. The reasons behind the formation of the Si_2_N_2_O remain to be answered. The Fourier inverse transformation diagram in Figure 10e,f clearly shows that the grain boundary region between©(C) and Si_2_N_2_O, Ti (C, N) © BN(C), included about four atomic layers with a thickness of ~2 nm and no impurity phases existed on the grain boundary. This structure of Ti (C, N), SiC, Si_2_N_2_©and BN(C) is isolated from each other, and the absence of impurity phases at the grain boundaries blocks the long-range diffusion of atoms and prevents the abnormal growth of each grain.

**Figure 11:**
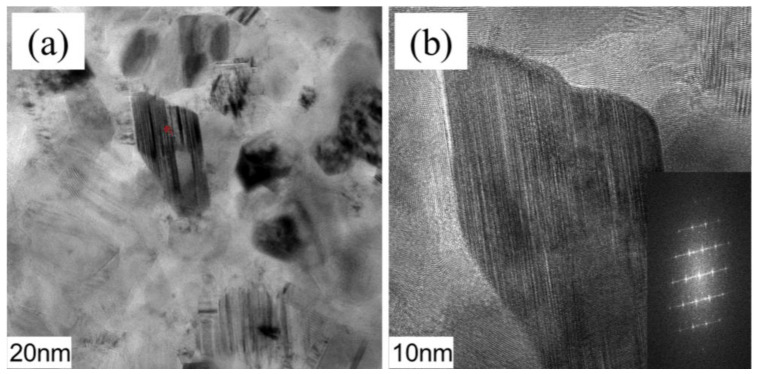
Detailed information concerning SiC grains in SiBCN-15 wt.% Ti bulk ceramics: (**a**) TEM image and the corresponding EDS, red symbol is the position of EDS analysis; (**b**) HRTEM image with the IFFT image.

As shown in Figure 11, substantial bright and dark streaks, indicative of stacking faults, were found to be distributed in some SiC grains. Furthermore, EDS analysis suggested the limited solution of some B, N, and Ti atoms in SiC, as shown in Table 2. Apparently, due to the limited solution of external atoms, the SiC lattice has been distorted irregularly [31].

**Figure 12:**
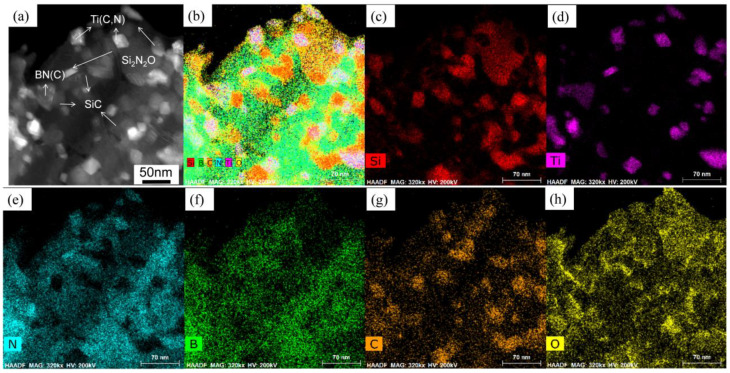
The element distribution mappings of the SiBCN-15 wt.% Ti bulk ceramics: (**a**) STEM-HAADF image; (**b**–**h**) corresponding to Si, B, C, N, Ti, O mappings.

To have a more accurate understanding of the detailed structure information, the Si, B, C, N, Ti, and O element mappings are exhibited in Figure 12. As indicated, the B and N elements were almost distributed in the same position. Some C elements were distributed in the B and N, but their distribution was relatively more concentrated in the Si-enriched area. Additionally, Ti elements were distributed in B, C and N regions but were more in C- and N-enriched regions and less in B-enriched regions. The O mapping almost overlapped with N and Si mappings, suggesting the distribution of Si_2_N_2_O in the ceramic matrix.

**Figure 13:**
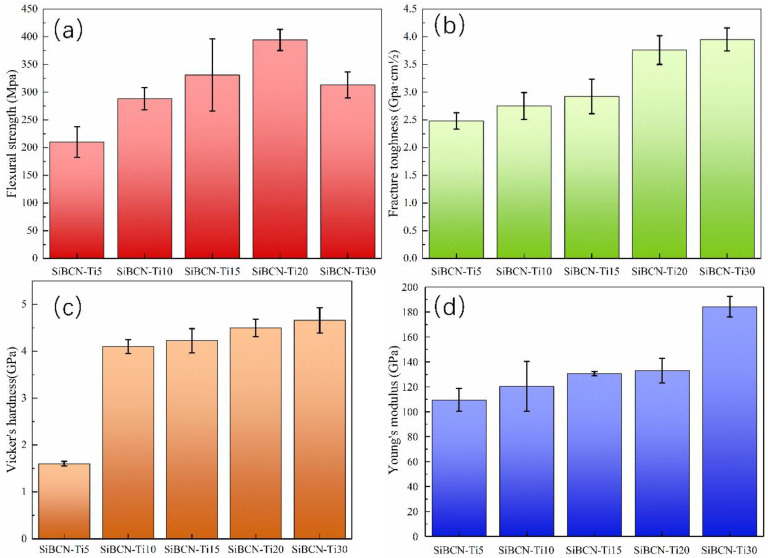
Mechanical properties of as-sintered SiBCN-Ti bulk ceramics: (**a**) flexural strength; (**b**) fracture toughness; (**c**) Vickers hardness (**d**) Young’s modulus.

The flexural strength, fracture toughness, elastic modulus, and Vickers hardness of the as-sintered SiBCN-Ti bulk ceramics are presented in Figure 13. As exhibited, the fracture toughness, Young’s modulus and Vicker’s hardness of the resulting bulk ceramics increased with the increasing addition of Ti. The SiBCN-20 wt.% Ti bulk ceramics obtained the highest flexural strength of 394.0 ± 19.0 MPa; furthermore, SiBCN-30 wt.% Ti bulk ceramics individually exhibit the best fracture toughness of 3.95 ± 0.21 GPa·cm^1/2^, Vickers hardness of 4.7 ± 0.27 GPa, Young’s modulus of 184.2 ± 8.23 GPa, and bulk density of 2.85 g/cm^3^. However, except for the bending strength gradually increased first and subsequently deceased with increasing Ti content, the fracture toughness, Vickers hardness, and elastic modulus of the bulk ceramics increased continuously with the increasing Ti content, which was closely related to their phase composition, phase scale and distribution, and microstructural/morphological change. On the one hand, the increasing content of Ti(C, N) with higher strength and desirable interfacial bonding favors mechanical property improvements. On the other hand, the formed turbostratic BN(C) were reported [20] to effectively inhibit the anomalous growth of each grain in the SiBCN matrix. Last but not least, the addition of Ti promoted the sintering ability of the composite powder, finally leading to the densification of SiBCN-Ti(C, N) bulk ceramics, thereby obtaining higher mechanical performance.

## 4. Conclusions

In this work, various SBCN-Ti ceramics were prepared through a combination of mechanical alloying and hot-pressing sintering techniques. After the first-step mechanical alloying of BN and Ti for 30 h, amorphous phases containing B, N and Ti elements with few TiN have been achieved. After the second-step mechanical alloying of all of the residual raw materials, amorphous phases with few nanosized ~5–7 nm Ti(C, N) have been obtained. During hot-press sintering, the main phases of BN(C), SiC and Ti(C, N), and a small amount of Si_2_N_2_O were found. The as-formed BN(C) enwrapped both SiC and Ti(C, N) and effectively inhibited the rapid growth of SiC and Ti(C, N). The flexural strength, fracture toughness, Vickers hardness and elastic modulus of the as-sintered SiBCN-Ti bulk ceramics increased continuously with the introduction of Ti. The SiBCN-20 wt.% Ti bulk ceramics obtained the highest flexural strength of 394.0 ± 19.0 MPa; however, the SiBCN-30 wt.% Ti bulk ceramics exhibited the optimized fracture toughness of 3.95 ± 0.21 GPa·cm^1/2^, Vickers hardness of 4.7 ± 0.27 GPa, Young’s modulus of 184.2 ± 8.2 GPa, and bulk density of 2.85 g/cm^3^.

## Figures and Tables

**Table 1:** Density and mechanical properties of SiBCN ceramics and their composites reproduced from Refs. [20,22,23,24].

Samples	Density (g/cm^3^)	Flexural Strength (MPa)	Elastic Modulus (GPa)	Fracture Toughness (MPa m^1/2^)	Vickers Hardness (GPa)
SiBCN [20]	–	313 ± 5	136 ± 18	3.31 ± 0.02	4.2 ± 0.5
SiBCN-MWCNTs	2.58	462 ± 50	115 ± 2	5.54 ± 0.60	5.1 ± 0.2
SiBCN-graphene	2.44	135 ± 8	150 ± 3	5.40 ± 0.63	2.4 ± 0.1
SiBCN-Zr	4.11	400	252	3.16	9.6
SiBCN-Al [22]	2.90	527 ± 10	222 ± 28	5.25 ± 0.20	11.6 ± 0.5
SiBCN-AlN	2.74	416 ± 147	148 ± 8	4.08 ± 1.18	6.4 ± 1.2
SiBCN-SiCf [24]	2.57	284 ± 18	184 ± 11	2.78 ± 0.14	–
SiBCN-HfC-TaC	–	344.1	–	4.52	–
SiBCN-LaB2 [23]	~2.75	372 ± 15	166 ± 18	4.2 ± 0.1	6.4 ± 0.2

**Table 2:** The composition of SiC grain at the red dot in Figure 11a obtained by EDS analysis.

Elements	Weight %	Atomic %
B(K)	23.82	33.59
C(K)	18.06	22.92
N(K)	20.59	22.41
O(K)	1.92	1.83
Si(K)	35.21	19.11
Ti(K)	0.37	0.11

## Data Availability

The data used to support the findings of this study are available from the corresponding author upon request.
